# Towards TB Elimination in Aotearoa/New Zealand: Key Informant Insights on the Determinants of TB among African Migrants

**DOI:** 10.3390/tropicalmed3020044

**Published:** 2018-04-22

**Authors:** Emmanuel Badu, Charles Mpofu, Panteá Farvid

**Affiliations:** 1 Department of Public Health, Auckland University of Technology (AUT), 90 Akoranga Drive, Northcote 0627, New Zealand; 2 Department of Interprofessional Health Studies, Auckland University of Technology (AUT), 90 Akoranga Drive, Northcote 0627, New Zealand; charles.mpofu@aut.ac.nz; 3 Department of Psychology, Auckland University of Technology (AUT), 90 Akoranga Drive, Northcote 0627, New Zealand; pani.farvid@aut.ac.nz

**Keywords:** tuberculosis, African, migrant, determinants, elimination

## Abstract

Migrants living in low incidence countries, including New Zealand (NZ), are disproportionately affected by tuberculosis (TB). This foreign-born group poses important challenges to achieving the national TB elimination targets. Thus, the aim of this study was to contribute to the understandingof factors that influence the incidence of TB among African migrants living in NZ. We employed a semi-structured interview approach to explore the perceptions of NZ-based African community leaders, health professionals and a non-governmental TB support organisation about the wider determinants of TB. The findings, though not completely generalizable, suggest that many NZ-based Africans endure a difficult process of integration, perceive themselves as least susceptible to TB and have low awareness about available health services. Furthermore, the cost of general practitioner (GP) services, mistrust of health professionals, TB stigma and the NZ immigration policy were indicated as important barriers to TB services. Strategies to address TB among migrants must therefore be more holistic and not be centred on a fragmented approach that overemphasises the biomedical approaches, as the incidence of TB is more likely the outcome of a complex interplay of several underlying factors.

## 1. Introduction

Tuberculosis (TB) remains an important public health threat globally. In 2015 alone, an estimated 10.4 million people were diagnosed and 1.8 million died from the disease [1]. Though a curable disease, it caused more deaths globally than HIV/AIDS and was the leading cause of death from infectious diseases in 2015 [1]. TB affects every region of the world, although a high level of disparity exists between and within countries. The existing inequalities in TB burden follow the prevailing socio-economic gradient that runs from the bottom to the top of the socio-economic spectrum; low income countries report more cases than high income countries, and the most deprived individuals report more cases than the least deprived within countries [2].

In low incidence countries, defined as countries reporting less than 100 TB cases per 1,000,000 population [3], the introduction of the WHO’s post-2015 TB strategy has reinvigorated public health action against TB. Many of these countries are seeking innovative approaches and tools to rapidly reduce TB incidence, which has levelled off since the turn of the century [4,5,6]. For instance, recent publications from Oman [7] and Cyprus [8] have demonstrated that TB elimination is feasible and would require that countries cover the core intervention areas within the elimination framework. 

Despite a number of documented benefits, immigration is one enduring challenge to TB elimination efforts in low incidence countries, including New Zealand (NZ). Foreign-born persons living in these countries contribute the highest proportion of all TB cases notified annually [5]. In NZ for instance, the foreign-born population is estimated to constitute about a quarter (25.2%; 1,001,787 people) of the population [9], yet they report the highest proportion (77.6%) of TB cases [10]. 

Globally, an estimated one billion people are migrants with the number expected to rise [11]. The sharp increase in recent arrival of migrants to Western countries, especially to Europe in the past year, has been met with some strong anti-migrant sentiments in certain host countries [12,13]. These anti-migrant sentiments could influence the marginalisation of migrants in their new countries. For instance, previous studies in NZ have noted that portraying migrants through public discussions in the media as people whose presence contributes to diseases, puts pressure on jobs and infrastructure, and threatens dominant cultural values, strengthens the feeling of ‘otherness’ and marginalisation that migrants experience in all aspects of their life including employment, education, housing, health and other social services [14,15]. 

As indicated, the reinvigorated drive to eliminate TB in low incidence countries has seen more emphasis on improvement of available clinical tools, particularly, around pre-arrival and post-arrival screening and treatment of latent TB infection (LTBI) [16]. In most cases, addressing the underlying factors are perceived as complementary rather than the fulcrum around which efforts to eliminate TB should revolve [17,18]. While improvement in existing tests are necessary and would contribute to minimising TB reactivation among high risk groups [19], there are debates about the accuracy of the existing screening tests to predict progression from LTBI to active TB [19,20,21].

In spite of the growing global concern to address the existing inequities in TB burden, especially among migrant communities in low incidence countries [22], there have not been many studies exploring the determinants of TB among persons born in sub-Saharan Africa living in NZ (generally referred to as Africans in this study). Yet, TB rates within this group (19.4 per 100,000 population) remain relatively high compared to persons born in NZ (2.3 per 100,000 population) [10]. To our knowledge, the only study on TB among Africans in NZ focused on the Somali refugee community [15]. In that study, Lawrence described the resettlement process as challenging and stressful for many of her participants. She reported that TB was highly stigmatised among her study population and that perceptions about health and illness were informed by participants’ experiences in their home country, Somalia. Although the study contributes to understanding how TB develops among Somalian refugees in NZ, it does not answer the broader question of what it is about being a sub-Saharan African migrant that leads to TB, as it does not reflect the different sub-Saharan African migrant groups in NZ. Other migrant ethnic specific TB studies in NZ among Asian [23,24] and Pacific peoples [25] have reported limited knowledge about TB, negative attitudes, high levels of stigma and treatment adherence as some of the common challenges to TB control. These studies demonstrated that the vulnerabilities to TB were not the same for the different communities within Auckland and recommended further work to explore these underlying determinants of TB.

In general, there is a paucity of published literature on the determinants of TB among Africans living in Western countries. The available studies, mostly from the UK, suggests that African migrants may have varied knowledge levels about TB and may delay in seeking help as initial symptoms of TB are more likely to be ignored [26,27,28,29]. Other factors such as stigma [26,28], mistrust of healthcare professionals [26,27,28,30] and challenges with language [31] have also been shown to influence the diagnosis and treatment of TB. In NZ, knowledge about the available services and the organisation of the healthcare system [32] influence people’s decision to seek help. 

In addition to these factors are people’s living conditions. Poverty has long been associated with TB and remains an important risk factor for TB infection and TB disease [33,34]. It is closely linked to overcrowding, which directly increases the risk of exposure to the TB bacilli [2]. Similarly, poverty influences nutrition and other lifestyle choices of individuals that may compromise the body’s defence mechanisms, leading to individuals progressing from TB infection to disease [35,36]. 

The aim of this study was therefore to explore how these factors identified from the literature—individual, social, economic and structural—may interact to influence TB incidence among African migrants in NZ, based on key informant perspectives. Understanding these determinants is critical to developing effective public health interventions for TB elimination.

## 2. Materials and Methods 

The material for this paper comes from a larger mixed methods study, which employed a sequential explanatory method in two phases. The first phase described the epidemiology of TB, whilst the second explained the phase one findings by exploring in-depth perspectives of study participants. The results presented here are based on the latter. 

### 2.1. Participants

In line with the aim of the study, we purposively sampled 15 individuals of whom nine agreed to be interviewed. The sampling frame for this study included all leaders of the different African communities in Auckland (as proxy respondents for the migrant community), TB health professionals (both public health and clinicians), and staff from the only charity organisation that provides support for people with TB living in Auckland. The varied experiences and perceptions from the key stakeholders in TB control provided rich data to adequately answer the research question. The nine participants interviewed were above 18 years, had the necessary understanding of TB services and the African community, were available to participate, were articulate and could express their thoughts clearly to help in answering the research questions (see Table 1 for participant demographic detail). 

Participants were recruited through an advertisement sent through publicly-available email addresses of African community leaders. Emails of healthcare professionals were solicited through personal contacts. A semi-structured interview approach was employed. All interviews were conducted in person using a one-on-one format, lasted about 45 minutes to one hour, were audio-recorded with permission of the participants, and subsequently transcribed after each interview. 

### 2.2. Analysis 

The interview data were analysed using thematic analysis. Thematic analysis has been defined by Braun and Clarke [37] as ‘a method for systematically identifying, organising and offering insight into patterns of meaning (themes) across a data set’ (p. 57). In this study, a hybrid thematic analysis, involving both inductive (data-driven) and deductive (theory- or literature-driven) approach, adopted from Braun and Clarke, was used [38]. The analysis was conducted in NVivo 11 software for Windows, computerised software for management of qualitative data. The coding process was iterative and begun after recorded interviews were transcribed and loaded into the analytic software. The codes were entered as nodes in NVivo and the text or extracts from the transcripts were coded by matching the text to the codes. The other codes that were identified inductively from the coding process were assigned to new nodes. The connections between the different codes, how they relate and their prevalence (number of times used by participants) within the data set were considered in identifying and categorising them into themes, which were further grouped into higher level clusters. 

### 2.3. Ethical Approval

The study received ethical approval from the Auckland University of Technology ethics committee (reference 16/128). 

## 3. Results

The themes identified from the analysis were categorised into five higher-level clusters: integration/settlement, individual level factors, economic, social, and structural factors. 

### 3.1. Settlement/Integration

African community leaders reported that the transitional process of migrating to a new country and resettling was daunting and stressful for many Africans:

I think what I found that is different is actually the cultures, the way of doing things, the processes that can be quite daunting if you’re coming from Africa.

*(CL01)*

This notion of a difficult transition process was further elaborated by the health professionals. They explained that many migrants including Africans faced difficult challenges, especially within the first few years of arrival, which influences the reactivation of previously-acquired latent TB infections:

The first two years after a migrant arrives in New Zealand is the most vulnerable time for the TB recurrence and that must be in my view a lot to do with stress and with the attack on the immune system that stress causes, and I mean that’s a whole multi-layered issue but you know, getting housing, and getting employment and getting people into work in the area that they’re skilled at and I don’t think we do particularly well.

*(HP01)*

One common challenge mentioned throughout the interviews was employment. Participants indicated that many Africans with higher educational qualifications were unlikely to find jobs in relevant industries or sectors of their training. In most cases their professional overseas degrees were not recognised in NZ:

A lot of people who’re professionals have resorted to what they call D3 jobs, which is basically dirty, difficult and demeaning. For example, when I was in Zimbabwe I was an executive manager but my very first job in New Zealand I was a toilet cleaner of all jobs, nobody wanted to employ me.

*(CL02)*

The community leaders described a common occurrence within the African migrant community, where professionals such as doctors, nurses, lecturers, and accountants have resorted to doing unskilled and odd jobs to secure some income and to survive. Doing such difficult unskilled jobs have consequences on the physical and psychological wellbeing of people. They are demeaning, according to this extract, and could lead to loss of self-esteem or self-worth as their previous status is lost. 

Other settlement process challenges mentioned as sources of stress to some new Africans related to language and the lack of awareness about available services:

Most of them don’t speak English very well and for that they try to limit themselves in terms of interacting or looking for where to go and seek the support.

*(CL04)*

What they find a little difficult is they don’t know where services are and they don’t know how to access some services.

*(CL01)*

Proficiency in the English language was indicated to inhibit the interactions of some Africans with people in their new society and created difficulties in finding jobs, accommodation, seeking healthcare and other services.

### 3.2. Low Perceived Susceptibility

Community leaders believed they were not at risk of acquiring TB in NZ:

I don’t consider myself to be at risk of TB in NZ here because from what I know TB is quite common back home in Africa but in NZ TB isn’t that common compared to the rate in Africa.

*(CL02)*

I don’t think I’m at risk.

*(CL04)*

I think it’s in places where probably there is poverty and that knowing that we don’t have that kind of poverty in New Zealand that means this kind of infections are not here either, the individual Africans don’t have it.

*(CL01)*

The leaders perceived TB as a disease for the poor, common in underdeveloped countries and so could not be acquired in a ‘rich’ country (NZ). This misconception was shared by all community leaders and might reflect the general view among Africans. Living in NZ was perceived to offer some form of protection and so the leaders seemed not to be particularly worried about such diseases. This notion may not be different from the mainstream NZ community, as some believed that TB had died out. For instance, it was a frequent occurrence during this study for both African migrants and persons of other ethnicities to ask in conversations with community members if there were actual cases of TB in NZ.

### 3.3. Economic Factors

Participants believed that while the financial and general living conditions of Africans were appreciable, the majority of them were of the low socioeconomic status:

I’ll say may be 60% of Africans living in NZ are on low income and the households in which they live, like a three-bedroom house will have about eight or nine people living in the house and most of the houses are housing NZ so it’s very damp and cold houses.

*(CL03)*

Community leaders’ perception of the living conditions of their members is supported by the 2013 census data, which points to an African group that is somewhat deprived socioeconomically. The African community leaders reported that although NZ was better than their own countries of origin, some of their members struggled economically. They explained that the poverty experienced by some of their members was in part due to the lack of information on the social support services available, the skills needed to access these services, and to compete in the new society. They further noted that the pressure on family income increased with the high cost of housing and rent, and general living expenses. 

This perception that TB commonly affected the poor was corroborated by the staff from the TB charity organisation:

TB generally attacks people who live in cold damp houses which often communities who’re financially challenged tend to be living in. Poorer accommodation with poor heating and poor curtains.

*(SO01)*

This participant, with several years of experience working for an organization that provides support for people living with TB, enumerated some risk factors that were common for persons with TB who required support from her organization. She noted that the most common support offered by her organization to families with TB were heating accessories as most of their clients lived in poorly ventilated and insulated homes.

### 3.4. Social Factors

Participants shared their opinion on how social factors impacted TB among African communities. Stigma was repeatedly mentioned by participants. They noted that TB was hidden among the African community. It was less frequently spoken about and persons with TB were more likely not to disclose to other family members due to the shame associated, and the fear of stigmatisation and isolation:

If someone knows from the community they may gossip about it or they may just try to withdraw themselves and that person can find themselves isolated.

*(CL04)*

This is certainly from what I’ve seen from the refugees from African backgrounds, having TB is deeply hidden not just from community members but from other family as well.

*(HP03)*

In addition, participants noted that the feeling of being labelled by other groups within their new society as the people with TB, HIV and other diseases contributed to some Africans concealing such diseases to reject the label:

I think too it’s linked to, as a saying, that sense of being picked on, you know, because I’m an African therefore I’ll have TB and I’m vulnerable to have HIV and every other disease. So, I think there’s a sense of westerners labelling as well.

*(HP01)*

The health professional believed that some Africans may simply refuse any contact with health professionals to avoid being labelled or to reinforce that their community was free from TB.

While the self-imposed feeling of guilt and shame associated with TB maybe pronounced among persons from close-knit communities such as the African communities, participants noted that people in general regardless of their ethnicity were uncomfortable finding out that they have TB:

Recently we diagnosed TB in a doctor he was of European decent and I remember that person was very upset when they were diagnosed not so much about having TB or worried about the treatment but was very worried about the effect it might have on the staff members where they worked if they found out.

*(HP04)*

This was linked to the notion that they might have passed the disease to other persons unknowingly and so they become more concerned about what others might think of them when they find out about their disease. 

Within the African communities, the strong perception that TB is linked with HIV, and that HIV is the underlying cause of TB was noted by participants as an important contributor the high stigma attached to TB:

TB is getting closer to HIV you know is the same level that we Africans consider TB even though we know TB can be cured but HIV cannot be cured but still people are too scared contracting that disease.

*(CL03)*

### 3.5. Structural Factors

Factors associated with the accessibility and delivery of TB services were identified as important determinants of TB among Africans living in NZ. These factors were perceived to have a direct impact on TB prevention, diagnosis and successful treatment. 

Within the conversations was a sense of mistrust between some Africans and their health professionals:

Most of us Africans are not really happy with the health system like when we visit our doctors.

*(CL04)*

Some of the doctors in New Zealand are a bit ignorant when it comes to some of the tropical diseases.

*(CL02)*

The leaders were convinced that some doctors in NZ lacked experience with tropical diseases. They believed that this influenced their ability to suspect cases early on, which sometimes led to delays in diagnosis.

Participants unanimously agreed that the immigration policy presented challenges to the early diagnosis and treatment of TB among migrants in NZ:

People who’re unfortunate to be diagnosed of TB while they’re here are often very fearful about what will happen to their immigration status particularly if they’re a visitor or on short-term permit.

*(HP04)*

We’ve some multidrug resistance TB cases almost to the end of their treatment, and then they say you’ve got to leave which seems very foolish and shortsighted.

*(HP02)*

An overstayer also will feel scared seeking for help because they know that if the doctor finds out that he or she’s an overstayer the doctor might call the immigration people to come and arrest him or her and deport him or her.

*(CL03)*

They explained that the lack of clarity and the inconsistency in the implementation of the immigration rules, as to who gets to stay or leave because of TB, made it difficult for health professionals to advise their clients appropriately. Health professionals were concerned about what they perceived as their clients’ anxiety about their immigration status to the point that they seemed less worried about their own health. They cited examples of people who abandoned their treatment some months into it, requesting their doctor to allow them some time to stay off TB treatment especially when they had a few months to submit their visa applications. 

Other barriers including difficulty in navigating the health system, lack of active community engagement and money were mentioned. Participants reported that the initial cost of accessing primary services pushed many to defer seeking treatment, prioritising resources for other more pressing needs:

The money as well, the financial side because going to see the GP costs money.

*(CL03)*

Cost is a barrier for many families here.

*(SO01)*

## 4. Discussion

This study, to the best of our knowledge, is the first to elucidate the determinants of TB among persons born in sub-Sahara Africa living in NZ. The findings from this study suggest that African migrants can experience difficult challenges during the process of integration into their new environment, which may influence the activation of previously acquired TB infection. This finding is consistent with that of Henrickson and his colleagues who, in their study on the knowledge, attitudes, behaviours and beliefs about HIV among black Africans living in NZ, reported that participants experienced difficulties in adapting to the host culture [39]. Moving to a new country requires adoption of new behaviours, values and language [40]. This process of acculturation, adapting to the new culture while maintaining one’s own, might lead to stress and risky health behaviours [40,41]. Lawrence observed in her study of Somali refugees in NZ, that most of her participants still grieved over their separation from families/friends and the loss of familiarity with their natural environment [15]. The sense of isolation from the wider society of the host country, the loss of ones’ previous status and the feeling of disappointment for some who had hoped for a better life upon arrival but found living in their new home to be more difficult than anticipated, compounds the stress on people [15,41]. 

African community leaders commonly embraced other perspectives to explain the causes of TB. Most of these perspectives related to the socio-economic circumstances of individuals including poor housing, poor nutrition, and overcrowding. A common misconception among African community leaders was that TB was not present in their new setting. Such low perceived susceptibility is consistent with existing literature [27]. While enough evidence about the link between TB and poverty exists, such strong perceptions, as identified in the current study, might lead African migrants to perceive themselves as unlikely to develop TB and are likely to be misinterpreted and unreported [42]. A possible explanation for the low perceived susceptibility might be that most participants were unfamiliar with the concept of latent TB, which could progress to TB disease at a later stage. It could also be due to the lack of awareness campaigns within the communities. The study found that services were constrained by resources to undertake such programmes, which affirms the common notion that TB is less prioritised in most low incidence countries [4].

An important finding from this study was a strong perception among community leaders about the link between TB and HIV. TB was commonly perceived as a precursor to HIV, and that any individual diagnosed with TB could have an underlying disease, most likely HIV. The findings suggest that TB within the African community may attract a double stigmatisation from both HIV and TB, as negative stereotypes about HIV are transferred to TB [43]. Previous studies have shown that HIV is highly stigmatised within the NZ African community [39] and this might reasonably explain the high sense of fear towards TB demonstrated by the community leaders interviewed in this study [43]. The implication for practice is that, interventions to address TB stigma among Africans in NZ would need to simultaneously focus on misconceptions about HIV. 

The study identified the NZ immigration policy as a significant barrier to TB elimination efforts. In Henrickson and colleagues report, they identified the existing immigration laws as a ‘source of distress’ for their participants [39]. In this study, it was noted that the policy contributed to non-reporting of TB symptoms as persons were fearful of deportation [11]. While individual knowledge about symptoms of TB could positively influence health decision, concerns about one’s immigration status has the potential to prevent actual behaviour. Porter, for example has argued that the recent introduction of a formalised process to allow the Health and Social Care Information Centre (NHS Digital) to transfer demographic data to the UK Home Office Immigration Enforcement Team was counterproductive and a major setback to TB elimination efforts, as most migrants, especially illegal migrants, would be deterred from seeking healthcare services for fear of deportation [6,12]. The immigration policy might be playing a more significant role in TB control efforts in NZ and would require further study to assess its impact.

The findings are somewhat limited due to the non-inclusion of Africans diagnosed with or recently cured of TB. Their participation could have provided personal and deeper insights into treatment-related challenges. Again, the findings must be applied with caution as study participants were purposively sampled and so do not represent the entire African community.

## 5. Conclusions

The research reported here came from an exploratory qualitative project examining an under-researched topic. Although the methodology used means we were not focused on generalizable results, the outcome provides initial insights into what factors may influence TB incidence in NZ. The findings demonstrate that the early years of settlement/integration post-arrival can be extremely difficult for many African migrants and with that an increased vulnerability to TB disease and other health conditions. Furthermore, the findings suggest that African migrants are likely to experience economic challenges, especially within the first few years of arrival which may influence many to defer visits for care and live in low standard housing. The findings also suggest the NZ immigration policy can inhibit migrants on temporary visas with symptoms suggestive of TB from seeking help and completing their treatment due to the threat of deportation or denial of visa renewal. 

This study recommends that a national TB strategy with specific targets and ring-fenced funding be developed to guide TB elimination efforts in NZ. In addition, a coalition of organisations led by the public health TB control team is required to intensify advocacy for more resources, as in the case of HIV, cancers and other high priority diseases. The adoption of policies that will prevent voluntary or forced deportation of people until they have successfully completed treatment is needed to facilitate early diagnosis and treatment. To advance understanding of the determinants, a survey with a sample size that reflects the African population is warranted. 

## Figures and Tables

**Table 1:** Participant Demographic Characteristics.

Participant	Sex	Category	Occupation
CL01	Female	Community leader	HIV advocate
CL02	Male	Community leader	Programme manager
CL03	Male	Community leader	Social worker
CL04	Male	Community leader	Programme coordinator
HP01	Female	Healthcare worker	Programme manager
HP02	Female	Healthcare worker	Medical officer of health
HP03	Female	Healthcare worker	Public health nurse
HP04	Male	Healthcare worker	Respiratory physician
SO01	Female	Support organization	Manager
